# Formulation of ciprofloxacin-loaded oral self-emulsifying drug delivery system to improve the pharmacokinetics and antibacterial activity

**DOI:** 10.3389/fphar.2022.967106

**Published:** 2022-10-04

**Authors:** Asghar Ali Asghar, Muhammad Akhlaq, Aamir Jalil, Abul Kalam Azad, Junaid Asghar, Muhammad Adeel, Ghadeer M. Albadrani, Amin A. Al-Doaiss, Mohamed Kamel, Ahmed E. Altyar, Mohamed M. Abdel-Daim

**Affiliations:** ^1^ Faculty of Pharmacy, Gomal University, Dera Ismail Khan, Pakistan; ^2^ Department of Pharmaceutics, Faculty of Pharmacy, Bahauddin Zakaria University, Multan, Punjab, Pakistan; ^3^ Faculty of Pharmacy, MAHSA University, Jenjarom, Malaysia; ^4^ Institute of Chemical Sciences, Gomal University, Dera Ismail Khan, Pakistan; ^5^ Department of Biology, College of Science, Princess Nourah Bint Abdulrahman University, Riyadh, Saudi Arabia; ^6^ Biology Department, Faculty of Science, King Khalid University, Abha, Saudi Arabia; ^7^ Department of Medicine and Infectious Diseases, Faculty of Veterinary Medicine, Cairo University, Giza, Egypt; ^8^ Department of Pharmacy Practice, Faculty of Pharmacy, King Abdulaziz University, Jeddah, Saudi Arabia; ^9^ Department of Pharmaceutical Sciences, Pharmacy Program, Batterjee Medical College, Jeddah, Saudi Arabia; ^10^ Pharmacology Department, Faculty of Veterinary Medicine, Suez Canal University, Ismailia, Egypt

**Keywords:** ciprofloxacin, pseudo-ternary phase diagram, self-emulsifying drug delivery system, oral bioavailability, solubility

## Abstract

This study aims to increase the aqueous solubility of ciprofloxacin (CPN) to improve oral bioavailability. This was carried out by formulating a stable formulation of the Self-Emulsifying Drug Delivery System (SEDDS) using various ratios of lipid/oil, surfactant, and co-surfactant. A pseudo-ternary phase diagram was designed to find an area of emulsification. Eight formulations (F1-CPN–F8-CPN) containing oleic acid oil, silicone oil, olive oil, castor oil, sunflower oil, myglol oil, polysorbate-80, polysorbate-20, PEO-200, PEO-400, PEO-600, and PG were formulated. The resultant SEDDS were subjected to thermodynamic study, size, and surface charge studies to improve preparation. Improved composition of SEDDS F5-CPN containing 40% oil, 60% polysorbate-80, and propylene glycol (Smix ratio 6: 1) were thermodynamically stable emulsions having droplet size 202.6 nm, charge surface -13.9 mV, and 0.226 polydispersity index (PDI). Fourier transform infra-red (FT-IR) studies revealed that the optimized formulation and drug showed no interactions. Scanning electron microscope tests showed the droplets have an even surface and spherical shape. It was observed that within 5 h, the concentration of released CPN from optimized formulations F5-CPN was 93%. F5-CPN also showed a higher antibacterial action against *S. aurous* than free CPN. It shows that F5-CPN is a better formulation with a good release and high antibacterial activity.

## 1 Introduction

Ciprofloxacin (CPN) is a quinolone with fluorine at position 6 of the naphthyridine ring (Verderosa et al., 2017). The structure–activity relationship data show that fluorine atoms help broaden the activity spectrum against both Gram-negative and Gram-positive pathogens (Hu et al., 2017). CPN is a synthetic fluoroquinolone antibiotic with broad-spectrum activity against Gram-positive and Gram-negative bacteria. The primary target of fluoroquinolones is the bacterial enzyme DNA gyrase or topoisomerase II. CPN is generally well-tolerated; safe and adverse effects vary significantly depending upon their physicochemical properties (Satpati et al., 2016). Low aqueous solubility and limited oral bioavailability have always been the major problems in translating various lead components into clinical use (Yoo et al., 2010). SEDDS are an isotropic combination of natural and synthetic oils, non-ionic surfactants, or hydrophilic solvents and co-solvents/surfactant combinations (Patel et al., 2011). Lipid-based formulations of drugs such as SEDDS are very efficacious for enhancing drug solubility (Wu et al., 2006). SEDDS are isotropic combinations of oils of either natural or synthetic origin, surfactants, and co-surfactants (Shafiq et al., 2007). These preparations best ease drug absorption via an intestinal lymphatic pathway, which protects the drugs from hepatic first-pass metabolism (Czajkowska-Kosnik et al., 2015). As SEDDS reach the GIT, these systems can form fine O/W emulsion or micro-emulsions after little agitation. These preparations will disperse in the gastrointestinal tract, and the motility of the GIT provides sufficient agitation for self-emulsification (Elnaggar et al., 2009). The advancement in combinatorial and medicinal chemistry leads us to develop new molecules with varied pharmacological activity (Gershanik and Benita, 2000). However, with the increasing number, the number of hydrophobic drugs is also increasing, leading to insufficient absorption through the gastrointestinal tract (GIT) and limited bioavailability (Bharti et al., 2019). The current study aims to formulate a stable SEDDS formulation with different proportions of lipid surfactants and co-surfactant-surfactants to increase water solubility and improve the bioavailability of CPN orally. Previous studies increased the relative bioavailability of the highly hydrophobic drug acyclovir to 12.78 through SEDDS formulation (Ljiljana et al., 2018). The nanoparticles facilitate drug passage through the mucosal membrane in the GIT. It could facilitate sustained and rapid effects of drugs. Its anhydrous nature makes it easy to fill in gelatin capsules and shows good patient compliance (Zidan et al., 2007). Self-micro-emulsifying drug delivery systems (SMEDDS) usually produce emulsions of globule sizes ranging from 100 to 300 nm, and self-nano-emulsifying drug delivery systems (SNEDDS) produce clear microemulsions of globule size <100 nm. Compared to the sensitive and metastable emulsions in dispersion, SEDDS are more physically stable and easy-to-manufacture formulations.

Therefore, lipid formulations show dissolution rate-limited absorption. These formulations give an enhanced level of bioavailability, resulting in more reproducible blood–time profiles (Zupancic et al., 2016). These formulations had attained attention when Neoral^®^ (cyclosporine-A) and Norvir^®^ (ritonavir) SEDDS got success commercially (Nazzal et al., 2002). CPN showed antibacterial activity by targeting DNA gyrase or topoisomerase II bacterial enzymes. CPN is normally considered a safe and well-tolerated drug (Hu et al., 2017). In the existing contribution to ameliorate aqueous dissolvability parallel with oral bioavailability, SEDDS of the CPN base was formulated with different proportions of lipids, surfactants, and co-surfactants (Shafiq et al., 2007). The resultant formulation was comprehensively characterized by various techniques such as stability studies, droplet size, and zeta potential.

## 2 Materials and methods

The materials CPN base was provided as a gift sample by Feroze-Son Pharma (Nowshera 24,110, Pakistan). Tween 80 and Tween 20 were purchased from Sigma Aldrich (Saint Louis, Missouri 63,101, United States). Oleic acid oil, silicone oil, olive oil, castor oil, and sunflower oil were obtained from Gaziantep (Gunedogu Anadolu 34,810, Turkey). Mygliol oil, PG, polyethene oxide 200, polyethene oxide 400 (Dow Chemical Ltd, Bangkok 10,110, Thailand), and polyethene oxide 600 were obtained from Ahad International Pharmaceutical, Dera Ismail Khan, 29,050, Pakistan.

### 2.1 Solubility studies

Solubility studies were performed to screen excipients to confer compatibility and safety and facilitate formulation manufacturing. Various oils, surfactants, and co-surfactants, including oleic acid oil, olive oil, castor oil, sunflower oil, mygliol oil, T-80, T-20, PG, PEG200, PEG400, and PEG600, were used individually to check the drug solubility. An excess drug quantity was mixed in a vial with a 2-ml mixture of oil, surfactant, and co-surfactant on a vortex mixer (Fischer-Scientific, United Kingdom) to calculate the solubility properties of CPN in various oils, surfactants, and co-surfactants. The mixture was kept at 25 ± 2 °C in a thermo mixer (Medsinglong Co. Ltd., Guangdong, China), and the time point was checked for equilibrium solubility. Samples were taken at 2, 6, 12, 18, and 24 h. All samples were centrifuged (Hunan Labwe Scientific Instruments, United Kingdom) at 10 × 1000 rpm for 15 min, and the supernatant was collected and filtered using a 0.22-µm membrane filter, and the samples were measured at 277 nm by a UV–Vis spectrophotometer (Hitachi U-1800, Japan). In UV–Vis spectrophotometer analysis, the same ratio of oil and methanol (50:50) was used to avoid any background oil absorbance auto zero (Shafiq et al., 2007).

### 2.2 Pseudo-ternary phase diagram construction

From the result studies of the solubility parameters mentioned above, the selection was made for oil surfactants and co-surfactants to construct a pseudo-ternary phase diagram. The phase diagram was formulated for all surfactant and co-surfactant mixture. Oil and Smix proportions differ from 1/9 to 9/1 for the graph of the pseudo-ternary phase. Distilled water was added drop-per-drop to the mix. An observation was made to monitor the change from a clear to a turbid solution (Czajkowska-Kosnik et al., 2015).

The quantity of water added was measured by the weight difference between the oil and surfactant mix from pre and post-addition of water. Percentage composition of all was calculated, and Chemix software version 3.60 (Arne Standnes, Bergen 5,003, Norway) was used to plot the pseudo-ternary phase diagram. The pseudo-ternary phase diagram exposed a stable emulsification area for every Smix.

### 2.3 SEDDS preparation

The surfactant and co-surfactant mixture concentrations of between 30 and 60% showed stable nano-emulsion areas. Therefore, the formulations were prepared in the same range (i.e., 30%–60%), with four formulations in every phase diagram. 25 mg of the drug were added into 100 mg of the oil phase and gyre well using a vortex mixer. An adequate quantity of Smix was added to the oil drug mixture for 10 min and mixed well by homogenization. In all eight formulations, the oil content was kept unchanged, while the quantity of Smix was different, as shown in Table 1.

**TABLE 1:** Composition of different formulations.

Batch No	Weight of compositions (mg)					% Compositions (w/w)			
	Drug	Olive oil	Smix1	Smix2	Total wt. of batch	Olive oil	Smix1	Smix2	Total
F1	25	100	41.25	---	166.5	70	30	---	100
F2	25	100	45.38	---	170.38	60	40	---	100
F3	25	100	100	---	225	50	50	---	100
F4	25	100	150	---	275	40	60	---	100
F5	25	100	---	41.25	166.5	70	---	30	100
F6	25	100	---	45.38	170.38	60	---	40	100
F7	25	100	---	100	225	50	---	50	100
F8	25	100	---	150	275	40	---	60	100

S_mix1_: 6:1 ratio of T80 and PEG, 600. S_mix2_: 6:1 ratio of T80 and PG.

### 2.4 Physical characterization

#### 2.4.1 Thermodynamic studies

Formulation centrifugations were carried out for 15 min at 6 × 1,000 rpm, and only formulations without phase separation were selected for the freeze–thaw stress test. Selected formulations were subjected to three freeze–thaw sets performed at 40 °C, room temperature, and 20 °C. The formulations were retained for 48 h at each temperature (Nagasreenu et al., 2020).

#### 2.4.2 Identification of self-emulsification time

Dissolution apparatus II (USP) was used to study self-emulsification time. 500 ml of distilled water was placed in each dissolution vessel stirring at 50 rpm, 37 ± 2 °C. Furthermore, 100 µL of the SEDDS formulation sample was added to the apparatus, and the time of emulsion formation represents the self-emulsification time (Czajkowska-Kosnik et al., 2015).

#### 2.4.3 Robustness to dilution

All formulations were mixed with water, acid buffer pH 1.20, and phosphate buffer pH 6.80 (1,000, 100, and 50 times). Visual observation rejected all formulations on the basis of emulsion breakage (Elnaggar et al., 2009).

#### 2.4.4 Cloud point measurement

A cloud point is the temperature where a clear emulsion becomes turbid. For this, 1 ml of all formulations was taken and mixed with 200 ml of water, and the temperature was slowly increased by using a water bath. By visual inspection, cloud point test results of stable preparations were noted in triplicates (Gershanik and Benita, 2000; Elnaggar et al., 2009).

### 2.5 Droplet size and zeta potential analysis

The size of droplets after SEDDS emulsification is very important in SEDDS development. SEDDS with smaller droplet size and greater surface area showed high dissolution and absorption (Gershanik and Benita, 2000). The selected SEDDS formulations were diluted with water 100 times and mixed well with an ultrasonic homogenizer (E60 H, Elma Hans Schmidbauer & Co, Singen, Germany) for at least one minZeta sizers were used to analyze optimized SEDDS formulations for size, PDI, and zeta potential (ZS90, Malvern Instrument, London, United Kingdom). For analysis, glass cuvettes were used and the back scattering angle was 175°(Bharti et al., 2019).

### 2.6 Morphology studies

De-ionized water was used to optimize the formulation by diluting it 50 times. The obtained sample was positioned on a glass slide to give tiny films. The slides were freeze-dried after dehydrating the thin film and advanced for superficial morphology studies *via* an EVO235 Scanning Electron Microscope (SEM) MA15/18 EDS: 51N1000 (Carl Zeiss/EDAX. Inc.) (Ljiljana et al., 2018).

### 2.7 Fourier Transform Infra-Red (FT-IR) studies

Possible interactions between the API and all excipients used in the formulation design were evaluated by infrared spectroscopy with Fourier transformation. Pure CPN and optimized formulation were analyzed within 4,500–450 cm^−1^. Zinc-selenide ATR (attenuated total of 58 references) mode fixed at 16 scans per sample was used to analyze samples (Zidan et al., 2007).

### 2.8 *In vitro* drug release

The dialysis tube method (Float-a-lyser^®^, MWCO = 100 kDa) was used to investigate *in vitro* drug release by following the method of Zupancic et al. with slight modification (Zupancic et al., 2016). A stock solution of CPN (0.17 mg/ml), F2-CPN, and F5-CPN (0.1 w/v) was constituted in a preheated 50 mM phosphate buffer pH7.4 at 37 ± 2°C. Then, 1.0 ml of CPN base stock solution and 1 ml of each formulation were filled in dialysis tubes. Every sample was dialyzed at 300 rpm at 37 ± 2°C, by stirring a thermo-mixer in 25 ml of phosphate buffer. 0.1-ml aliquots were taken from the medium at an interval of 0, 1, 2, 3, 4, 5, and 6 h, and an equal volume of 50 mM pH 7.4 phosphate buffer was used as a replacement. Fluorescence spectroscopy was used to determine the amount of CPN after background correction through excitation at wavelength 335 nm and emission at the wavelength of 420 nm with a micro-plate reader (Infinite ™ M200, Austria).

### 2.9 SEDDS diffusion study

Friedl et al. described a method where a slight modification was used to gauge the mucus-infusing properties of SEDDS (Friedl et al., 2013). 24-well Transwell^®^ plates were superficially enclosed with 60 mg goat intestinal mucus used for diffusion studies. For the homogenous thickness of mucus in each well, agitation by shaking on a shaking board (Vibramax-100, Heidoolph instrument, Schwabach, Germany) at 37 ± 2°C, 300 rpm up to 15 min, was carried out. Subsequently, in the acceptor chamber containing 750 µL of 50 mM pH 7.4 phosphate buffer at 37 ± 2°C, the donator compartment was filled with 1/4th mL of the SEDDS emulsion having CPN. SEDDS formulations were prepared by adding 100 mg of F2-CPN and F5-CPN in 1 ml of 50 mM pH 7.4 phosphate buffer. Incubation of plates at 3 37 ± 2 °C with 20 rpm was carried out on a shaking board, and samples of 100 µL were taken at 0,1,2,3, and 4 h and replaced with an equal amount of buffer. Permeated CPN was determined using fluorometric detection at an emission of 420 nm and an excitation of 335 nm.

### 2.10 Determination of the minimum inhibitory concentration

The National Committee for Clinical Laboratory Standards (NCCLS) guideline (NCCLSCALSI, 2012) for the broth microdilution method was used to determine the minimum inhibitory concentration (MIC). The MIC is the drug’s minimum concentration that entirely stops the organism’s growth in micron dilution. F2-CPN and F5-CPN emulsions and pure CPN solutions were prepared by standard two-fold dilution of a CPN concentration (ranging from 0.01 to 5µ) in sterilized water using a 96-well micro-titration plate (Greiner Bio-One, Germany). Positive control was prepared using pure CPN. Inoculum suspensions of *Escherichia coli* (ATC^®^ 2,592), *Pseudomonas aeruginosa* (ATC^®^ 2,785), and *Staphylococcus aureus* (ATC^®^ 2,921) were incubated first in sterilized water at 0.5 McFarland. Second, it was inoculated into Mueller–Hinton–Broth (MHB) with a final concentration of approximately 5 × 10^5^ CFU per mL. As a final point, 50-µL suspensions were inoculated to plates having a two-fold dilution of free CPN and CPN SEDDS formulations. The last antibiotic concentration was observed with the naked eye after incubating for 16–20 h at 37 ± 2 °C and quantified by 450 nm at a microplate reader (Bio Red Model 680 Microplate Reader).

### 2.11 Time-kill assay (TKA)

For the time-reliant bactericidal activity of CPN SEDDS, an assay to estimate the killing time was performed following Gruber et al., with slight modification (Martina et al., 2017). Formerly adjusted 0.5 McFarland *Escherichia coli*, *Pseudomonas aeruginosa*, and *Staphylococcus aurous* inoculums were added to preheated F2-CPN and F5-CPN formulations, pure CPN solution, and blank F2 and F5 formulations one-to-one. The CPN concentration used is shown in Table 2. Concluding inoculum concentration was attained by 1/250 dilutions for *E. coli* and 1/400 dilutions for *Pseudomonas aeruginosa* and *staphylococcus aurous* incubated for 1, 2, 3, 4, and 5 h at 37 ± 2 °C. For the sample, using an automatic spiral platter (WASP 2, Don Whitely and Shipley, United Kingdom), test inoculums were placed on Mueller–Hinton agar plates in triplicates. The incubation time of plates was 24 h at 37 ± 2°C, and then CFU was counted in numbers. As a negative control, inoculum suspension deprived of antibiotics in empty F2 and F5 emulsions was incubated in a pyrogen-free water/MHB solution (1:1v/v).

**TABLE.2:** Concentrations of CPN in F2-CPN, F5-CPN, and CPN solution were used to determine TKA against *E. coli*, *S. aurous*, and *P. aeruginosa*.

	CPN (µg/ml)		
Preparation	*Escherichia coli*	*Pseudomonas aeruginosa*	*Staphylococcus aureus*
**CPN**	0.6	4	4
**F2-CPN**	0.6	4	4
**F5-CPN**	0.6	6	4

### 2.12 Statistical analysis

All experiments were performed in triplicate. The two-sample t-test was carried out using Graph Pad Prism (ver. 5.01) for statistical data analysis and ANOVA. The Bonferroni correction (adjustment of probability values) was performed with *p* < 0.05. All values are stated as mean ± SD.

## 3 Results

### 3.1 Solubility study

**FIGURE 1:**
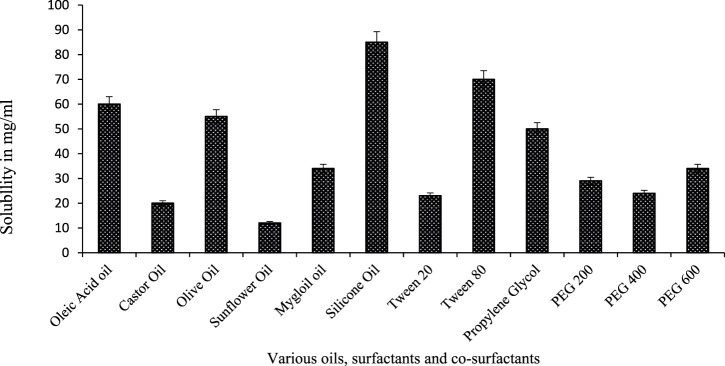
Drug solubility in various components (surfactants, oil, and co-surfactants), mean ± SD, *n* = 3.

Drug solubility plays a major role in the selection of formulation components. The drug stocking capacity of SEDDS directly depends on solubility in surfactants, oil, and co-surfactants (Shafiq et al., 2007). This work aims to compound a formulation with such components, which are safe, easily available, and with decent solubilizing capacity for CPN. The solubility of CPN in such components is given in Figure 1. Silicone oil, Tween 80, PG, and PEG showed maximum solubility of 85 mg/ml, 70 mg/ml, 50 mg/ml, and 30 mg/ml, respectively.

### 3.2 Pseudo-ternary phase diagram

**FIGURE 2:**
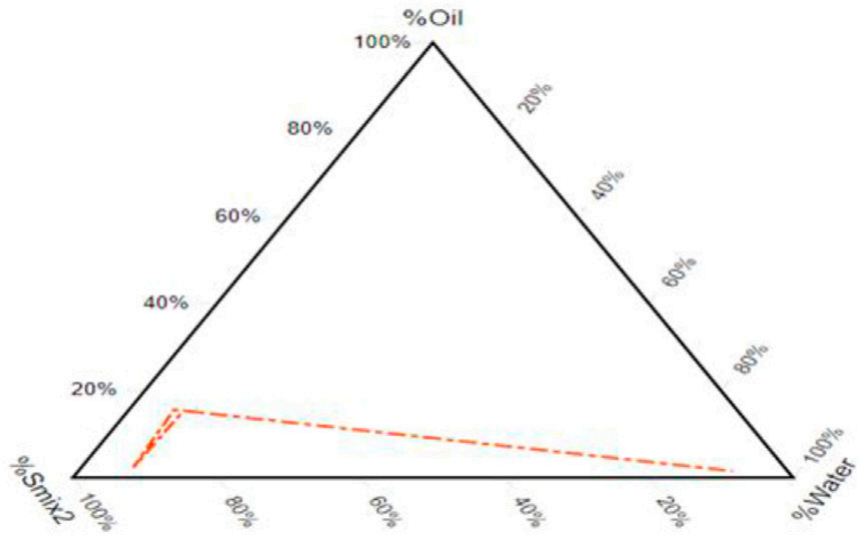
Pseudo-ternary phase diagram of oil–Smix2–water.

After component selection built on drug solubility, the resulting pseudo-ternary phase diagram showed a fixed proportion of surfactants and co-surfactants (Smix). A pseudo-ternary phase diagram of a system consisting of P.G./P.E.G. 600 and T-80 in a 1: 6 ratio is formed with a wide emulsifying area in which there is silicone oil. Figure 2 illustrates that the emulsifying area in the system surges with the increase in the mixture of surfactants. It is found in numerous research articles that enhancing the surfactant mixture decreases droplet size and vice versa (Czajkowska-Kosnik et al., 2015). It was observed that the self-emulsifying area is not significantly affected by adding the drug.

### 3.3 SEDDS preparation

Eight formulations (F1-CPN to F8-CPN) were formulated in this work. The composition of each formulation is given in Table 3. All formulations underwent physical characterization. The best formulation after physical characterization was retained for further work.

**TABLE 3:** Formulation composition name and amount.

Formulation No	Formulations weight					Formulation % weight			
	Drug (mg)	Silicone oil (mg)	Smix (mg)	Smix2 (mg)	Total weight (mg)	Silicone oil	Smix1	Smix2	Total
F1-CPN	25	100	41.25	---	166.5	70	30	---	100
F2-CPN	25	100	---	100	225	50	---	50	100
F3-CPN	25	100	100	---	225	50	50	---	100
F4-CPN	25	100	150	---	275	40	60	---	100
F5-CPN	25	100	---	41.25	166.5	70	---	30	100
F6-CPN	25	100	---	45.38	170.38	60	---	40	100
F7-CPN	25	100	45.38	---	170.38	60	40	---	100
F8-CPN	25	100	---	150	275	40	---	60	100

Smix1 6:1 ratio (Polysorbate 80 and PEG 600). Smix2 6:1 ratio (Polysorbate 80 and PG).

### 3.4 Physical characterization

#### 3.4.1 Thermodynamic studies

Thermodynamics studies were directed to evaluate the kinetic stability of the system. All formulations showed no phase separation and drug precipitations, passing the thermodynamic study test. Studies have shown that formulations are stable under these storage conditions (Nagasreenu et al., 2020).

#### 3.4.2 Self-emulsification time identification

An ideal SEDDS readiness would scatter rapidly and totally when it is weakened with little fomentation. It is characterized by numerous researchers that measured water dissemination into the oil and how it influences the self-emulsification rate (Czajkowska-Kosnik et al., 2015). The results of the test for self-emulsification time are displayed in Table 4.

**TABLE 4:** Percentage transmittance, emulsification time, clarity, cloud point, globule size, zeta potential, and polydispersity index data are presented as means (n = 3) ±SD.

Formulation	% Transmittance	Emulsification time (sec)	Clarity	Cloud point (°C)	Droplet size (nm)	PDI	Zeta potential
F1	80.2	163 ± 1	Turbid	60 ± 3.15	462.9 ± 7.3	1.02 ± 0.02	-9.11 ± 1.21
F2	90	96 ± 3	Clear	70 ± 3.6	320.6 ± 5.7	0.09 ± 0.01	-11.4 ± 0.87
F3	89	127 ± 1	Clear	67 ± 3.15	645 ± 6.3	1.03 ± 0.01	-3.46 ± 1.53
F4	83.9	143 ± 3	Turbid	63 ± 3.64	333.6 ± 5.7	0.30 ± 0.05	-2.64 ± 0.91
F5	91.3	91 ± 4	Clear	72 ± 1.68	202.6 ± 2.1	0.22 ± 0.03	-13.92 ± 1.25
F6	85.4	132 ± 2	Clear	65 ± 2.54	402 ± 5.4	0.42 ± 0.06	-5.52 ± 1.45
F7	81.3	123 ± 1	Turbid	66 ± 3.12	335 ± 5.1	0.39 ± 0.07	-11.92 ± 1.66
F8	87.3	103 ± 3	Clear	65 ± 4.10	369 ± 5.2	0.39 ± 0.06	-10.99 ± 1.19

Test results showed that expansion in the centralization of surfactants diminishes the time of emulsification, which is conceivably related to the fact that high surfactant fixation might expand the water entrance capacity to oil/water.

#### 3.4.3 Robustness to dilution

After administration, the GIT formulations go through infinite dilution with fluids that cause phase separation or drug precipitation. To avoid this difficulty, a dilution study at pH 1.2 and pH 6.8 and a preparation with better dilution ability will be stable in GI fluids. The results of this study in our research showed that all the formulations were stable after 50, 100, and 1,000 time dilutions with acidic buffer (pH 1.2) and phosphate buffer (pH 6.8), and the results are shown in Table 4.

#### 3.4.4 Cloud point measurement

A formulation’s cloud point is directly related to its resistance to environmental temperature. Surfactant solubility decreases with an increase in temperature (Nagasreenu et al., 2020). Good solubility of the surfactant in emulsion gives a stable emulsion. This study showed that only F2-CPN and F5-CPN showed a higher cloud point. A higher cloud point indicates better formulation stability in GIT. The result of each formulation is shown in Table 4.

### 3.5 Measurement of droplet size and zeta potential

**FIGURE 3:**
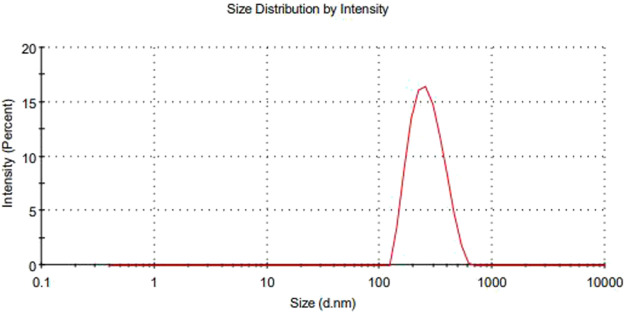
The range of droplet size of emulsion produced is shown *via* polydispersity index (PDI) values.

**FIGURE 4:**
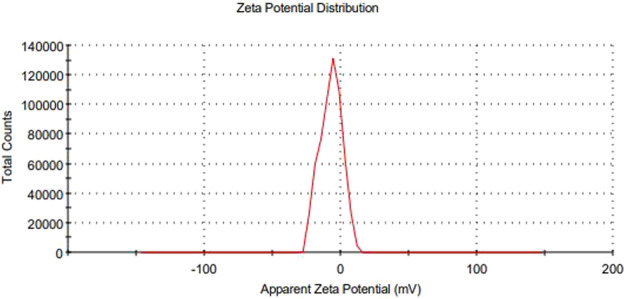
The zeta potential of particles.

The main concerns with SEDDS preparations are zeta potential and droplet size. Droplet size is a prime factor responsible for *in vivo* effects’ fate. All formulations experienced droplet size and PDI after 100-fold dilution, and F1-CPN, F3-CPN, F4-CPN, F6-CPN, F7-CPN, and F8-CPN formulations had droplet sizes greater than 400 nm, and only F2-CPN and F5-CPN formulations had droplet sizes of 320 and 202 nm, respectively, as shown in Table 4. The range of droplet size produced is shown via PDI. In this case, a good SEDDS formulation requires a low PDI, indicating that the droplet size is in a narrow range (Bali et al., 2010). As compared to other formulations, F2-CPN and F5-CPN formulations exhibited lower polydispersity. The results for the zeta potential tests are given in Table 4. Among all the formulations, only F2-CPN and F5-CPN formulations showed the -11.4 ± 0.87 and -13.38 mv zeta potential and were retained for further studies. The droplet size and zeta potential of F5-CPN are given in Figures 3, 4.

### 3.6 Morphology studies

**FIGURE 5:**
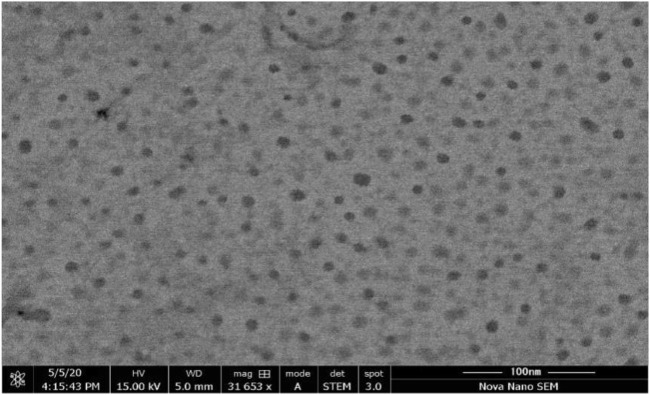
Morphology of F5-CPN by SEM

Morphology studies were performed by a scanning electron microscope to examine the morphological characteristics of the emulsion droplet with high resolution. SEM was performed for the optimized formulation F5-CPN, and the image is shown in Figure 5. The result indicated that the droplet size in the SEDDS formulation was smooth and sphere-shaped.

### 3.7 Interaction studies by infrared spectroscopy with Fourier Transformation

**FIGURE 6:**
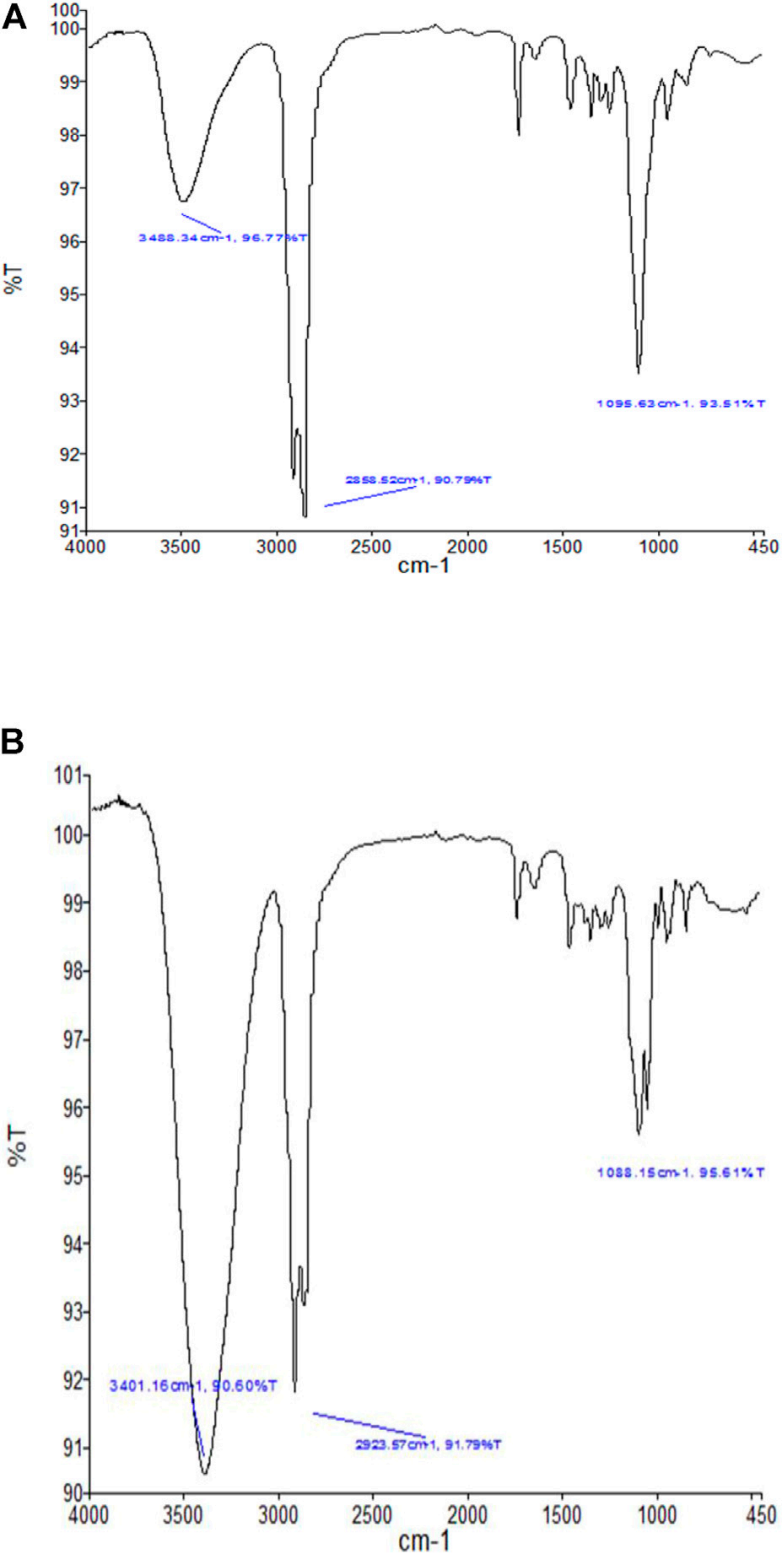
**(B)** FT-IR spectra of SEDDS containing CPN showing peaks at 1,088 cm^−1^, 2,923 cm^−1,^ and 3,401 cm^−1^.**(A)** FT-IR spectra of CPN showing characteristic peaks at 1,095 cm^−1^, 2,858 cm^−1^, and 3,488 cm^−1^.

The FT-IR was used to analyze the incorporation of CPN (a drug) in SEDDS. Moreover, the stability of CPN and its non-interaction with the excipients was confirmed by comparing the IR spectra of the pure drug and SEDDS containing CPN. The pure CPN FT-IR spectrum showed absorption peaks at 1,095 cm^−1^, 2,858 cm^−1^, and 3,488 cm^−1,^ while the FT-IR spectra of the SEDDS containing CPN showed its absorption peaks at 1,088 cm^−1^, 2,923 cm^−1^, and 3,401 cm^−1^. The peaks are similar to pure CPN, depicting no interaction between the excipient of SEDDS and CPN. Moreover, it confirms that CPN is well-incorporated into the oily droplets of SEDDS. The FT-IR spectra of CPN and SEDDS containing CPN are shown in Figures 6A, B.

### 3.8 I*n vitro* drug release

**FIGURE 7:**
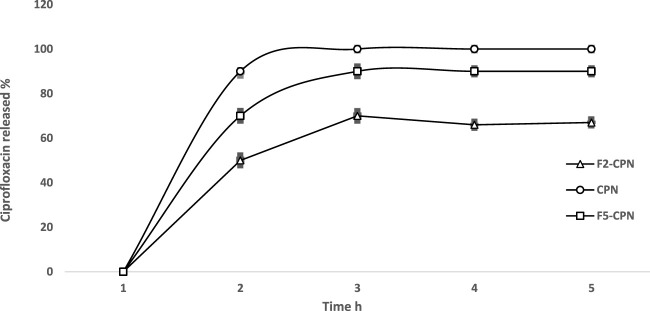
*In vitro* release formulations of CPN from solution [Δ], from SEDDS F2-CPN [□], and F5-CPN [o] by dialysis at 37 ± 2 °C. Data are presented as mean (n = 3) ± Std. Deviation (*p < 0.05).

For *in vitro* drug release, the dialysis membrane method was used, and the results are shown in Figure 7, giving a summary of CPN release from F2-CPN and F5-CPN. Free CPN was used as a control. The control was used to evaluate the membrane effect, if any, on drug release. CPN added into SEDDS was set as 100% for F2-CPN and F5-CPN in a distinct order. The study demonstrated quick release within 1 h, similar to the previous results (Lode et al., 1998). Slow drug release was also detected in both formulations. After 2 hours, 66.1 and 88.2% of CPN were released from both formulations, F2-CPN and F5-CPN, and after 5 hours, the concentration of the CPN released from F2-CPN and F5-CPN was up to 77.05 and 93.1%, respectively of the loaded drug. This result indicates that sufficient CPN concentration was achieved within a small time frame. CPN shows a concentration-dependent and concentration-dependent antibiotic effect, 21]. It is essential to attain an adequately high antibacterial concentration on the site for bactericidal action (Griesser et al., 2018).

### 3.9 SEDDS diffusion study

**FIGURE 8:**
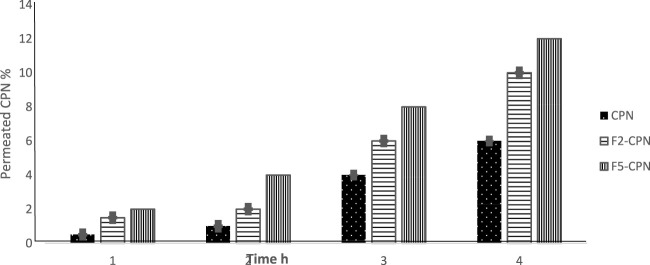
Intestinal mucus diffusion of indicated SEDDS formulations: F2 (red bars), F5 (green bars), and free ciprofloxacin (blue bars) *via* the Transwell^®^ setup method. Data are shown as mean (*n* = 3) ± SD (***p < 0.001 in comparison to free ciprofloxacin).

Diffusion studies of SEDDS for each formulation were performed on goat intestinal mucus. F2-CPN and F5-CPN exhibited a 1.6-fold and 2.3-fold higher permeation of CPN than free CPN in 4 h, as shown in Figure 8. F2-CPN and F5-CPN showed droplet sizes of 320 and 202 nm. The result indicates a size-dependent diffusion behaviour. The size-dependent diffusion behaviour of SEDDS, also reported by Friedl et al., was observed through the mucus layer (Friedl et al., 2013) Griesser et al., clearly highlighted a link between mucus permeation and droplet size. It was also demonstrated that liposomes and SEDDS with nanoparticles have significantly higher mucus permeation properties (Griesser et al., 2018). Accordingly, the result described above shows that CPN permeation is mostly connected to the mucus permeating properties of SEDDS.

### 3.10 MIC determination

Antimicrobial activity of CPN SEDDS, MIC was determined against (ATC^®^ 2,592), *Pseudomonas aeruginosa* (ATC^®^ 2,785), and *Staphylococcus aureus* (ATC^®^ 2,921), and all these strains proved a kin susceptibility to free CPN along with F2-CPN and F5-CPN. Results of the broth microdilution assay are shown in Table 5. Against *E. coli,* no significant difference between the antimicrobial activity of free CPN and F2-CPN was found. However, a slight increase in the MIC of F5-CPN was found compared to that of the free CPN. The MIC values of F2-CPN and free CPN are analogous to those of Gram-negative bacteria, *P. aeruginosa*. Meanwhile, the efficiency of F5-CPN is low, as shown in Table 5. In the case of *S. aurous,* F2-CPN, F5-CPN, and free CPN exhibited little difference in their antibacterial activity. Blank SEDDS showed no effect on each bacteria culture. In conclusion, these study outcomes showed that the antibacterial activities of free CPN and incorporated CPN in SEDDS are close to each other due to the better mucus-permeating properties of SEDDS. It is reasonable to assume that CPN SEDDS will have higher antibacterial activity than free CPN at the target site.

**TABLE 5:** *In vitro* action of free CPN and CPN incorporated in SEDDS against *E.coli, P. aeruginosa,* and *S. aureus.*

MIC (µg/ml)			
Formulation	*E.coli*	*P. aeruginosa*	*S. aureus*
Free CPN	0.03	0.305	0.399
F2-CPN	0.02	0.367	0.324
F5-CPN	0.040	0.670	0.342

### 3.11 Time kill assay

**FIGURE 9:**
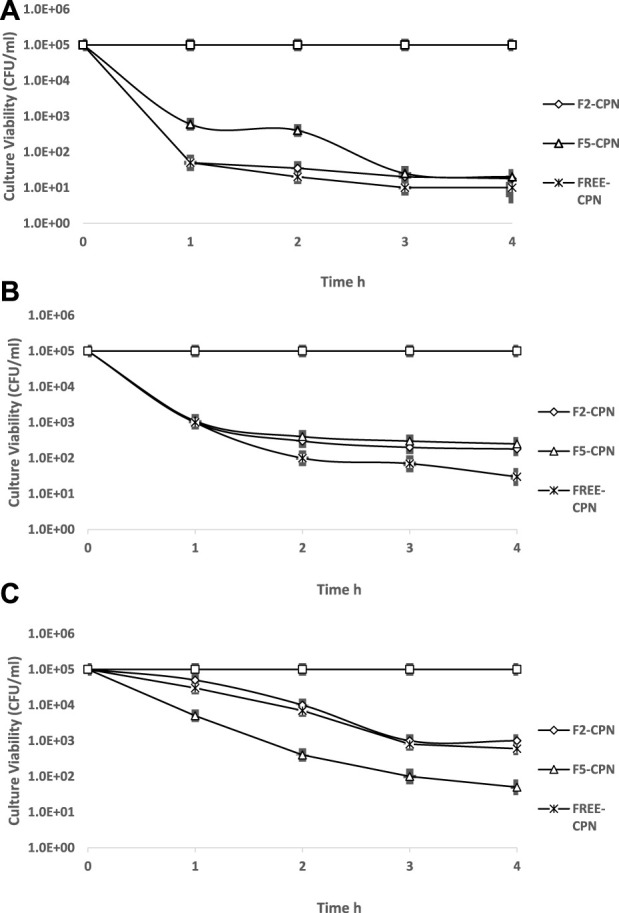
**(A)** *E. Coli* ATC®2592 by F2-CPN [♦] 0.5 μg/mL, F5-CPN [Δ] 0.5 μg/mL, free CPN [✕] 0.5 μg/mL, F2 [●] and F5[*]. Data are shown as a means (n = 3) ± SD (*p < 0.05, **p < 0.01, ***p < 0.001 in comparison to free CPN). **(B)** *P. aeruginosa* ATC®2785 by F2-CPN [♦] 3 μg/mL, F5-CPN [Δ] 5 μg/mL, free CPN [✕] 3 μg/mL, F2 [●] and F5[*]. Data are shown as a means (n = 3) ± SD (*p < 0.05, **p < 0.01, ***p < 0.001 in comparison to free CPN). Time kill assay of **(C)** *S. aureus* ATC^®^2,921 by F2-CPN [♦] 3 μg/ml, F5- CPN [ Δ ] 3 μg/ml, free CPN [**✕**] 3 μg/ml, F2 [●], and F5 [*] at 37 ± 2°C. Data are shown as a means (n = 3) ± SD (*p < 0.05, **p < 0.01, and ***p < 0.001 in comparison to free CPN).

In the time-kill assay, the time-dependent antibacterial action of CPN-SEDDS was linked to free CPN. As shown in Figure 9A, culture of *E. coli* showed an evaluated sensitivity to free CPN and F2-CPN, which brought an instant antibacterial action, dropping viability from 1.01×10^5^ and 1.07 × 10^5^ CFU/ml, respectively, to below 10^2^ CFU/ml within 1 hour. Contrary to that, the F5-CPN formulation displayed lower antibacterial activity with a reduction of the CFU/ml from 1.13 × 10^5^ to 10^2^ in 4 hours. Blank F2 and F5 formulations exhibited no antibacterial activity.

Figure 9 Time kill assay of (A) *E. Coli* ATC^®^2,592 by F2-CPN [♦] 0.5 μg/ml, F5- CPN [Δ] 0.5 μg/ml, free CPN [**✕**] 0.5 μg/ml, F2 [•], and F5 [*]. Data are shown as a means (*n* = 3) ± SD (*p < 0.05, **p < 0.01, ***p < 0.001 in comparison to free CPN).

According to Figure 9B, between free CPN and SEDDS formulations, a significant difference in antibacterial effect against *P. aeruginosa* was found. In 4 hours, F2-CPN reduced CFU/ml from 1.9 × 10^5^ to below 10^2^. At the same time, F5-CPN reduced from 1.5 × 10^5^ to 2.7 × 10^3^ in 5 hours, and blank F2 and F5 showed no antibacterial activity against *P. aeruginosa* culture.

Figure 9 Time kill assay of **(B)** *P. aeruginosa* ATC^®^2,785 by F2-CPN [♦] 3 μg/ml, F5- CPN [Δ] 5 μg/ml, free CPN [**✕**] 3 μg/ml, F2 [•], and F5 [*]. Data are shown as a means (n = 3) ± SD (*p < 0.05, **p < 0.01, and ***p < 0.001 in comparison to free CPN).

In Figure 9C, F2-CPN and F5-CPN formulations in the first 2 hours showed a killing rate similar to that of free CPN tested. After 5 hours, *S. aureus* became sensitive to F5-CPN. F2-CPN exhibited a significant difference at the *p* < 0.001 level compared to that of free CPN, while blank F2 and F5 formulations showed no antibacterial effect.

The delayed killing effect of F5-CPN against Gram-negative bacteria might be explained by MIC increase compared to free CPN. Consequently, a sufficiently high level was most probably achieved after 4 hours due to the concentration-dependent killing. In the case of Gram-positive bacteria, the improved permeating properties and high lipophilicity of SEDDS might improve permeation through murein, delivering the drug directly to the target site (Martina et al., 2017).

## 4 Discussion

The oral route of drug administration is the major route of drug delivery for disease treatment. About 40% of new chemical entities show low aqueous solubility, leading to low oral bioavailability. Low aqueous solubility and limited oral bioavailability have always been major issues in translating various lead components into clinics. CPN is a broad-spectrum synthetic fluoroquinolone antibiotic in the BCS IV group (Satpati et al., 2016). As drugs of this class have aqueous solubility problems, SEDDS are used as a delivery system in this study (Patel et al., 2011). SEDDS are an isotropic combination of natural and synthetic oils, non-ionic surfactants, or hydrophilic solvents and co-solvent/surfactant combinations. Lipid-based formulations of drugs such as SEDDS are very efficacious for enhancing drug solubility. SEDDS can produce fine oil in water emulsion after dilution in GI fluids with mild agitation provided by gastric mobility and provide a large interfacial area for drug partitioning between oil and water phases, increasing solubility rate and extent of absorption (Wu et al., 2006). The advancement in combinatorial and medicinal chemistry leads us to develop new molecules with varied pharmacological activity (Gershanik and Benita, 2000). However, with the increasing number, the number of hydrophobic drugs is also increasing, leading to insufficient absorption through the gastrointestinal tract (GIT) and limited bioavailability (Bharti et al., 2019).

To scheme the SEDDS preparations of the required physicochemica1 properties, the assortment of constituents is the utmost key portion. The solubility of a drug in different components will determine the drug loading capacity of SEDDS. Solubi1ity studies were performed to identify the oi1, surfactant, and co-surfactants to obtain maximum drug loading, and CPN showed maximum solubility in silicone oil, Tween 80, PG, and PEG. Pseudo-ternary phase diagrams of a system indicated that a mixture consisting of P.G./P.E.G. 600 and T-80 in a 1: 6 ratio formed with a wide emulsifying area. Numerous research articles claim that enhancing the surfactant mixture decreases droplet size and vice versa. In this case, the self-emulsifying area is not significantly affected by the addition of the drug (Czajkowska-Kosnik et al., 2015). The outcomes for an assortment of components demonstrate that the self-emulsifying properties of combinations with Tween 80 are superior, as emulsification is a rate-limiting step for drug absorption. The efficacy of CPN-SEDDS depends upon rapid self-emulsification. This study’s results indicate that the F5-CPN formulation has a short self-emulsification time.

The main concerns with SEDDS preparations are zeta potential and droplet size. Droplet size is a prime factor responsible for *in vivo* effects’ fate. The range of droplet sizes produced is shown *via* PDI. In this case, a low PDI is necessary for a good SEDDS formulation where the droplet sizes are in a narrow range (Bali et al., 2010). All formulations experienced droplet size and PDI after 100-fold dilution, and F1-CPN, F3-CPN, F4-CPN, F6-CPN, F7-CPN, and F8-CPN formulations had droplet sizes greater than 400 nm, and only F2-CPN and F5-CPN formulations had droplet sizes of 320 and 202 nm, respectively, as shown in Table 4. As compared to other formulations, F2-CPN and F5-CPN formulations exhibited lower polydispersity. Formulations F2-CPN and F5-CPN showed the -11.4 ± 0.87 and -13.38 mv zeta-potential, respectively. The morphology studies by SEM were performed for the optimized formulation F5-CPN. The result indicated that the droplets in the SEDDS formulation have a smooth surface and are sphere-shaped.

FT-IR results showed no interaction between the drug and excipients. Moreover, it confirms that CPN is well-incorporated into the oily droplets of SEDDS. The release studies of F2-CPN and F5-CPN demonstrated quick release within 1 h, similar to the results mentioned above. Then, slow drug release was detected in both formulations. After 2 hours, 66.1 and 88.2% of CPN were released from both formulations, F2-CPN and F5-CPN. After 5 hours, the concentration of the released CPN from F2-CPN and F5-CPN was up to 77.05 and 93.1%, respectively, of the loaded drug. So, sufficient CPN concentration at the objective site might be reached in a short time. The literature reports that CPN shows a concentration-dependent, antibiotic effect (Lode et al., 1998; Zhanel et al., 2002). It is essential to attain an adequately high antibacterial concentration on the objective site for bactericidal action (Griesser et al., 2018).

In this work, the results of the diffusion study showed that the droplet size for F2-CPN is 320 nm and 202 nm for the F5-CPN droplet. They demonstrated a 1.6-fold and 2.3-fold higher permeation of CPN than free CPN in 4 h. This is linked to the size-dependent diffusion behaviour of SEDDS. The size-dependent diffusion behaviour of SEDDS was also reported by Friedl et al. through the mucus layer (Friedl et al., 2013). Furthermore, Griesser et al. highlighted a link between mucus permeation and droplet size. It was also demonstrated that liposomes and SEDDS with nanoparticles show much higher mucus permeating properties. Accordingly, the result mentioned earlier shows that CPN permeation is mostly connected to the mucus permeating properties of SEDDS (Griesser et al., 2018). Antimicrobial activity against *E. coli* showed no significant difference between free CPN and F2-CPN. However, a slight increase in the MIC of F5-CPN was found compared to the free CPN. The MIC values of F2-CPN and free CPN are analogous to Gram-negative bacteria *P. aeruginosa*, while the efficiency of F5-CPN was low, as shown in Table 5. In the case of *S. aurous,* F2-CPN, F5-CPN, and free CPN exhibited little difference in their antibacterial activity. Blank SEDDS showed no effect on each bacteria culture. In conclusion, this study showed that the antibacterial activities of free CPN and incorporated CPN in SEDDS are close to each other due to the excellent mucus permeating properties of SEDDS. Thus, it is reasonable to assume that CPN SEDDS will have more antibacterial activity than free CPN at the action site. It is reported in the literature that CPN shows a concentration-dependent antibiotic effect (Lode et al., 1998; Zhanel et al., 2002). Last, in terms of bactericidal action, it is essential to attain an adequately high antibacterial concentration on the objective site (Griesser et al., 2018).

## 5 Conclusion

This study found that CPN base SEDDS comprise several ratios of surfactants, lipids, and co-surfactants formulated to improve aqueous solubility and oral bioavailability. The SEDDS formulations were characterized by physical stability, solubility studies, droplet size, zeta potential, PDI, and high dissolution rate as compared to CPN solution. The selected formulations exhibited good surface morphology and physicochemical properties. The surfactant and co-surfactant combination for a stable formulation is very important. The formulations were relatively stable at refrigeration for 3 months. It is clear from our study that developed liquid SEDDS would be good nanocarriers for delivering CPN orally. It has been proven that SEDDS is a good tactic to improve their dissolution and absorption for low soluble and/or low permeable drugs.

## Data Availability

The raw data supporting the conclusion of this article will be made available by the authors, without undue reservation.

## Abbreviations

CPN, ciprofloxacin; SEDDS, self-emulsifying drug delivery system; PEO, polyethylene oxide; PG, propylene glycol; PDI, polydispersity index; FT-IR, Fourier transform infrared; CPN, ciprofloxacin.
